# Testosterone modifies U-Shaped association of eGFR with all-cause mortality in Chinese female centenarians: a prospective cohort study

**DOI:** 10.1186/s12958-026-01529-w

**Published:** 2026-01-28

**Authors:** Zehao Zhang, Xiaowei Cheng, Zeyu Qu, Yue Niu, Zhe Feng, Weiguang Zhang, Ding Sun, Hao Li, Qiushi Wang, Miao Liu, Yali Zhao, Yao He, Guangyan Cai, Xiangmei Chen, Bin Wang, Yizhi Chen

**Affiliations:** 1 Department of Nephrology, Hainan Hospital of Chinese PLA General Hospital, Hainan Province Chen Xiangmei Academician Team Innovation Center for Kidney Diseases Research, Sanya, 572013 China; 2 Chinese PLA General Hospital (PLA Medical School), Beijing, 100853 China; 3 Senior Department of Nephrology, State Key Laboratory of Kidney Diseases, National Clinical Research Center for Kidney Diseases, Beijing Key Laboratory of Medical Devices and Integrated Traditional Chinese and Western Drug Development for Severe Kidney Diseases, Beijing Key Laboratory of Digital Intelligent TCM for the Prevention and Treatment of Pan-vascular Diseases, Key Disciplines of National Administration of Traditional Chinese Medicine (ZYYZDXK-2023310), Innovation Team and Talents Cultivation Program of National Administration of Traditional Chinese Medicine (ZYYCXTD-D-202402), Chinese PLA General Hospital, 100853 Beijing, China; 4 Department of Anti-Nuclear, Biological, Chemical Medicine, the Graduate School of Chinese PLA General Hospital, Beijing, 100853 China; 5 Central Laboratory, Hainan Hospital of Chinese PLA General Hospital, Sanya, 572013 China; 6 Institute of Geriatrics, Beijing Key Laboratory of Geriatric Comorbidity, National Clinical Research Center for Geriatrics Diseases, the Second Medical Center of Chinese PLA General Hospital, Beijing, 100853 China; 7 The Second School of Clinical Medicine, Southern Medical University, Guangzhou, 510515 China; 8 Sanya Nephrology Medical Quality Control Center, Sanya, 572013 China

**Keywords:** Centenarians, All-cause mortality, Estimated glomerular filtration rate (eGFR), Testosterone, Female

## Abstract

**Background:**

The relationship between kidney function and mortality in centenarians, particularly with respect to hormonal regulation, remains unclear. This study investigated the association between estimated glomerular filtration rate (eGFR) and all-cause mortality in female centenarians and explored the potential role of testosterone.

**Methods:**

Within the China Hainan Centenarian Cohort Study, 701 female centenarians (median age: 102 years) were enrolled. eGFR was calculated using the CKD-EPI 2009 creatinine equation. Restricted cubic splines (RCSs) and multivariable Cox proportional hazards models were employed to assess nonlinear associations. Likelihood ratio tests were used to evaluate the interaction effect of testosterone.

**Results:**

During a median follow-up of 31 months, 643 participants (91.7%) died. RCS analysis revealed a significant nonlinear association (P-overall < 0.001; P-nonlinear = 0.004) between eGFR and all-cause mortality, characterized by a U-shaped curve, with an inflection point of 60.65 mL/min/1.73 m² identified in the adjusted RCS model. Multivariable-adjusted Cox regression analysis showed that each 10-mL/min/1.73 m² increase in eGFR was associated with a 6.8% reduction in all-cause mortality risk (hazard ratio = 0.932, 95% confidence interval: 0.882–0.985, *P* = 0.012). Compared with centenarians with eGFRs of 45–<60 mL/min/1.73 m², those with eGFRs of ≤ 45 mL/min/1.73 m² had a 44.9% increased risk of all-cause mortality (hazard ratio = 1.449, 95% confidence interval: 1.176–1.785, *P* < 0.001) and a significantly shorter median survival time (26 months vs. 34 months, *P* < 0.001). Higher testosterone levels attenuated the association between eGFR and all-cause mortality, indicating a protective effect (P for multivariable nonlinear interaction = 0.022).

**Conclusion:**

In female centenarians, lower eGFR (< 45 mL/min/1.73 m²) is independently associated with all-cause mortality, and this association is modified by serum testosterone levels. These findings highlight testosterone as a potential modulator of the kidney function–mortality relationship in centenarians, although the causal relationship requires further investigation.

**Supplementary Information:**

The online version contains supplementary material available at 10.1186/s12958-026-01529-w.

## Highlights

Novel U-shaped association: For the first time, we report a significant U-shaped association between estimated glomerular filtration rate (eGFR) and all-cause mortality in a large prospective cohort of female centenarians.

Critical renal thresholds: eGFR values of 45 and 60 mL/min/1.73 m² were identified as critical inflection points.

Testosterone as an effect modifier: Serum testosterone levels significantly modify the eGFR–mortality relationship, representing a novel finding in extreme aging.

Protective role of testosterone: Higher testosterone levels attenuate the increased mortality risk associated with low eGFR, suggesting a protective effect in this unique female population.

Sex-specific insights: This study challenges traditional paradigms by highlighting the potentially beneficial role of testosterone in renal function and overall survival among the oldest-old women.

**Supplementary Information:**

The online version contains supplementary material available at 10.1186/s12958-026-01529-w.

## Introduction

The global demographic transition has entered an accelerated phase of population aging, with centenarians representing the fastest-growing demographic segment. According to projections by the United Nations Development Programme, the global centenarian population—estimated at 487,000 in 2015—is anticipated to undergo exponential growth during the 21st century, potentially exceeding 25 million by 2100 [1]. This demographic shift is particularly pronounced in China, where the number of centenarians surged from 35,800 in 2010 to more than 118,000 in the latest national census [2]. Centenarians (aged ≥ 100 years) exhibit unique health trajectories and physiological characteristics distinct from those observed in younger elderly populations (aged 65–99 years), presenting distinct challenges and opportunities for geriatric healthcare systems.

The renal aging process is characterized by glomerulosclerosis (resulting in a 40% loss of functional nephrons by 80 years of age) [3], interstitial fibrosis, and arteriolar hyalinosis. These pathological changes collectively lead to an exponential decline in renal functional reserve. The estimated glomerular filtration rate (eGFR) serves as the cornerstone biomarker for assessing kidney function and is routinely used in clinical practice for diagnosing and monitoring kidney disease progression. Substantial evidence has demonstrated that reduced eGFR is strongly associated with end-stage kidney disease (ESKD), all-cause mortality, and cardiovascular mortality [4], indicating that this parameter is an independent predictor of mortality risk in the general population. However, current epidemiological investigations have focused primarily on traditional elderly populations, while a critical gap remains in understanding the association between eGFR and mortality within the centenarian population, particularly among female centenarians.

A multinational meta-analysis encompassing 46 cohorts with 2,051,158 participants demonstrated that at equivalent eGFR levels, men presented significantly higher risks of all-cause mortality and cardiovascular mortality than women [5]. However, the underlying mechanisms for these sex-based differences in the associations between eGFR and adverse outcomes remain unknown. Recent animal studies indicate that the NFE2-related factor 2 (NRF2) antioxidant pathway is a crucial mechanism protecting proximal tubule cells against ferroptosis in female mice, and that this protective pathway is suppressed by high testosterone levels [6]. On the contrary, a longitudinal study by Laughlin et al. suggested that low endogenous testosterone concentrations may adversely affect cardiovascular disease risk in women [7]. This paradoxical evidence implies a potential “threshold effect” of testosterone in females and suggests that the modulatory role of testosterone in the association of renal function and all-cause mortality urgently requires further investigation, especially in female centenarians.

As an international observatory for geroscience in China, Hainan provides a unique vantage point for research on successful aging. Leveraging the China Hainan Centenarian Cohort Study (CHCCS), this study is the first to investigate two critical scientific questions: First, does a nonlinear association exist between eGFR and all-cause mortality among female centenarians? Second, does the testosterone level modify the predictive effect of the eGFR on mortality risk? The findings from this research will contribute to redefining the paradigm of kidney function assessment in extreme aging and may offer a novel biomarker feature set for personalized survival prediction.

## Participants and methods

### Study participants

This study adopted a prospective cohort design, with data derived from the CHCCS. The CHCCS is a population-based cohort study targeting individuals aged 100 years and older in Hainan, China, and it represents the largest single-center centenarian cohort in Asia. With the collaboration of relevant institutions in Hainan Province, a multidisciplinary research team (including clinicians, epidemiologists, and geriatric nursing specialists) conducted baseline surveys of all centenarians in Hainan between June 2014 and December 2016.

Initially, 1002 centenarians were enrolled. The following predefined exclusion criteria were applied:

- Male participants (*n* = 180) because this study focused on associations between female hormones and outcomes.
- Missing key variables (*n* = 121; missing creatinine values: *n* = 32, 3.9%; missing sex hormone values: *n* = 89, 10.8%).

**Fig. 1:**
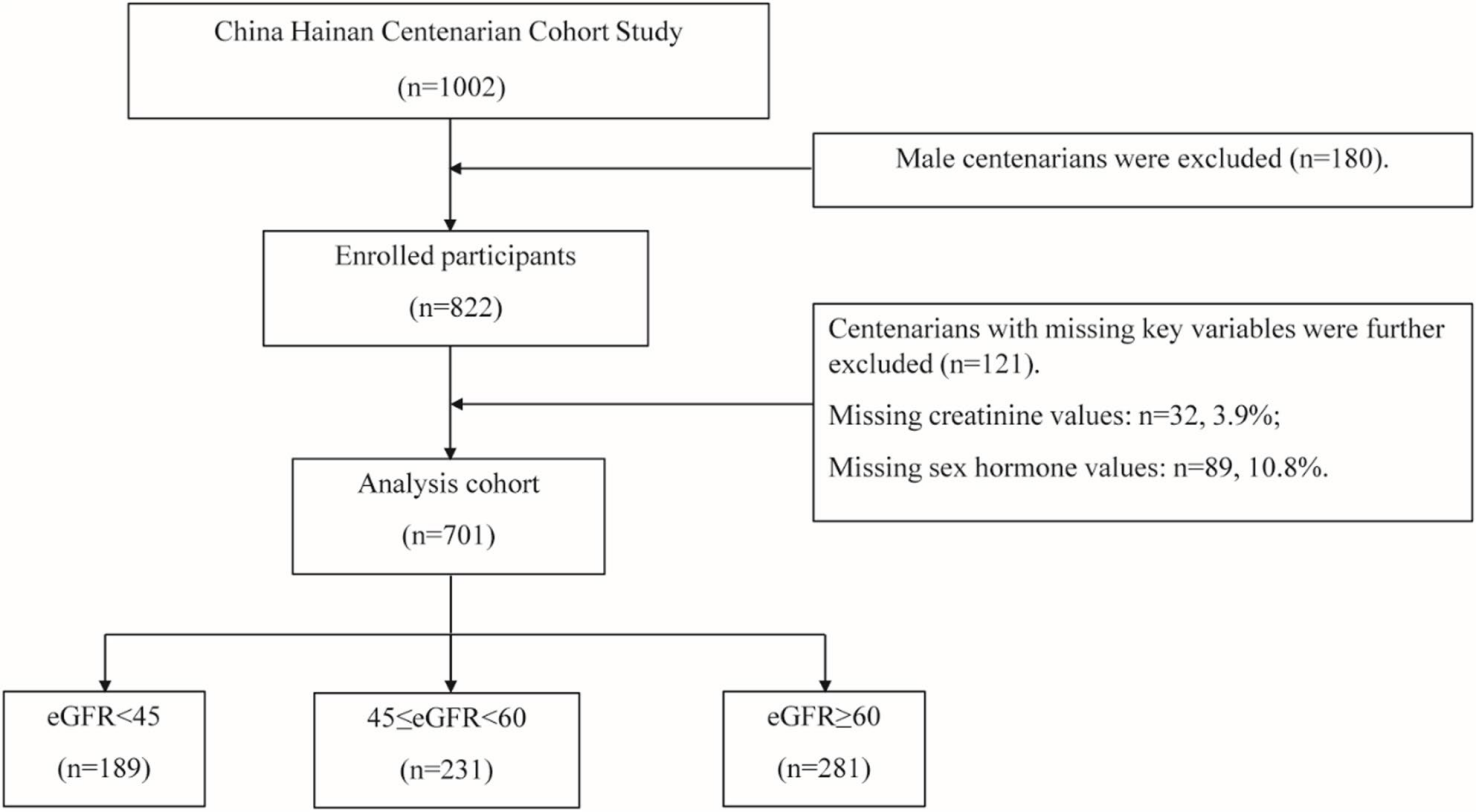
Flow chart of participant selection

Based on the Complete Case Analysis principle, the final analytical cohort consisted of 701 female centenarians (Fig. 1).

This study strictly adhered to the ethical principles of the Declaration of Helsinki of the World Medical Association. Written informed consent was obtained from all participants or their legal proxies. The study protocol was approved by the Ethics Committee of Hainan Hospital of the Chinese People’s Liberation Army General Hospital (No. 301HNLL-2016-01). This study was designed and reported in accordance with the STROBE guidelines for observational studies [8].

### Outcome definition

The primary outcome was all-cause mortality. To ensure data accuracy, dates of death were rigorously validated through a tripartite verification process:

- official records cross-referenced with the National Cause of Death Registration and Reporting Information System (China CDC).
- administrative confirmation by local civil affairs authorities.
- family verification via structured telephone interviews with next of kin.

Additionally, survival status was monitored monthly by the Hainan Provincial Civil Affairs Bureau through the pension disbursement system for individuals aged ≥ 80 years, ensuring real-time updates on participant vitality. All enrolled participants were prospectively followed from baseline (2014–2016) until the study endpoint on 31 March 2023. Notably, no participants were lost to follow-up.

### Baseline characteristics and covariates

#### Baseline characteristics

The collected baseline information included age, ethnicity, marital status, educational attainment, lifestyle factors (e.g., smoking and alcohol consumption), and medical histories of hypertension, diabetes mellitus, and coronary heart disease. Diagnoses of hypertension, diabetes mellitus, and coronary heart disease were based on self-reported data or the use of relevant medications. Hypertension was defined as systolic blood pressure of ≥ 140 mmHg and/or diastolic blood pressure of ≥ 90 mmHg. Diabetes mellitus was diagnosed according to the 1999 World Health Organization (WHO) criteria.

#### Renal function assessment

In this study, the eGFR was primarily calculated using the 2009 Chronic Kidney Disease Epidemiology Collaboration (CKD-EPI) equation. To verify the robustness of the results, additional repeated analyses and comparisons were performed. Five alternative equations were applied: the Berlin Initiative Study (BIS1) equation, the Full Age Spectrum (FAS) equation, the revised Lund–Malmö (RLM) equation, the equation from the European Kidney Function Consortium (EKFC), and the 2021 CKD-EPI equation. All eGFR values were derived from serum creatinine levels. Serum creatinine was measured using an enzymatic assay. Cystatin C was not collected in this study; therefore, only eGFR results based on serum creatinine are reported.

#### Laboratory assays

Serum phosphate levels (mmol/L) were measured using the standard phosphomolybdate ultraviolet assay. Serum levels of progesterone (nmol/L), prolactin (µg/L), estradiol (pmol/L), and testosterone (nmol/L) were measured via electrochemiluminescence immunoassay on a Cobas e602 analyzer. The reference ranges for postmenopausal women were as follows: estradiol, 18.4–505 pmol/L; testosterone, 0.101–1.42 nmol/L; progesterone, 0.3–2.5 nmol/L; and prolactin, 4.79–23.3 µg/L.

### Statistical analysis

Continuous variables conforming to a normal distribution are presented as mean ± standard deviation. Comparisons between groups were performed using independent-sample t tests or one-way analysis of variance. Continuous variables not conforming to a normal distribution are presented as median and interquartile range (IQR) and were analyzed using nonparametric tests (e.g., the Mann–Whitney U test or the Kruskal–Wallis H test). Categorical variables are presented as counts and percentages and were compared using the chi-square test. Correlations between parameters were assessed using Spearman’s rank correlation coefficient.

The proportional hazards assumption for eGFR was assessed prior to Cox regression analysis. The relationship between eGFR and all-cause mortality was evaluated using univariable and multivariable restricted cubic spline (RCS) analyses. Hazard ratios (HRs) and their 95% confidence intervals (CIs) for mortality risk associated with eGFR were estimated using Cox proportional hazards regression models. Survival distributions across eGFR subgroups were visualized using Kaplan–Meier curves and compared with the log-rank test. The effects of testosterone on the association between eGFR and mortality were assessed using the likelihood ratio test. Covariates were selected based on data availability, prior literature, and potential biological links between renal function and sex hormones. Multivariable models were adjusted for age, ethnicity, marital status, education level, body mass index (BMI), calf circumference, smoking status, alcohol consumption, hypertension, diabetes, coronary heart disease, serum phosphate, and sex hormones (testosterone, estradiol, progesterone, and prolactin). All statistical analyses were performed using R software (version 4.4.0). A two-sided P value of < 0.05 was considered statistically significant.

## Results

### Baseline characteristics

The CHCCS prospectively enrolled 701 female centenarians residing in Hainan Province, China (median age: 102 years; IQR: 101, 104). During the follow-up period, a total of 643 (91.7%) of the 701 female centenarians died from all causes. The median follow-up time was 31.20 (IQR: 15.20, 54.30) for the entire cohort. Baseline characteristics are detailed in Table 1. The participants’ median eGFR was 55.87 mL/min/1.73 m² (IQR: 44.13, 68.98). In accordance with the 2012 KDIGO Chronic Kidney Disease (CKD) guidelines, the participants were stratified into three groups: CKD stage 1–2(*n* = 281, 40%), stage 3a (*n* = 231, 33%), and stage 3b–5 (*n* = 189, 27%), 254 (90.4%) participants in stage 1–2, 204 (88.3%) in stage 3a, and 185 (97.9%) in stage 3b–5 died from all causes during follow-up. The prevalence of hypertension was 75.6%, while the prevalence rates of diabetes mellitus and coronary heart disease were 10.0% and 4.3%, respectively. Lifestyle characteristics indicated low-risk behaviors: 96.0% were nonsmokers, and 87.1% were nondrinkers. The median BMI was 17.74 kg/m² (IQR: 16.02, 19.81), which is below the normal range (18.5–24.9 kg/m²) defined by the WHO.

**Table 1:** Baseline demographic and clinical characteristics

Variable	Overall	[11.9,45)	[45,60)	[60, 96.1)	*P*
N, %	701 (100.0)	189 (27.0)	231 (33.0)	281 (40.0)	
Age, years	102 (101, 104)	102 (101, 104)	102 (101, 104)	102 (101,104)	0.030
Follow-up time, months	31.20 (15.20, 54.30)	25.80 (14.00, 45.50)	34.40 (16.60, 59.15)	31.30 (15.90, 54.90)	0.008
Death, %	643 (91.7)	185 (97.9)	204 (88.3)	254 (90.4)	0.001
Ethnicity					0.049
Han, %	621 (88.6)	162 (85.7)	200 (86.6)	259 (92.2)	
Other, %	80 (11.4)	27 (14.3)	31 (13.4)	22 (7.8)	
Marital status					0.470
Married, %	54 (7.7)	11 (5.8)	18 (7.8)	25 (8.9)	
Separation/Divorce/Widowhood, %	647 (92.3)	178 (94.2)	213 (92.2)	256 (91.1)	
Education					0.548
Illiterate, %	677 (96.6)	180 (95.2)	223 (96.5)	274 (97.5)	
Elementary school, %	22 (3.1)	8 (4.2)	7 (3.0)	7 (2.5)	
Junior high school and above, %	2 (0.3)	1 (0.6)	1 (0.5)	0 (0.0)	
Smoke					0.460
Never, %	673 (96.0)	181 (95.8)	221 (95.7)	271 (96.4)	
Past, %	20 (2.9)	4 (2.1)	9 (3.9)	7 (2.5)	
Now, %	8 (1.1)	4 (2.1)	1 (0.4)	3 (1.1)	
Drinking					0.197
Never, %	611 (87.1)	171 (90.5)	192 (83.1)	248 (88.3)	
Past, %	34 (4.9)	7 (3.7)	16 (6.9)	11 (3.9)	
Now, %	56 (8.0)	11 (5.8)	23 (10.0)	22 (7.8)	
Hypertension, %	530 (75.6)	147 (77.8)	181 (78.4)	202 (71.9)	0.170
Diabetes Mellitus, %	70 (10.0)	16 (8.5)	25 (10.8)	29 (10.3)	0.704
Coronary Heart Disease, %	30 (4.3)	9 (4.8)	11 (4.8)	10 (3.6)	0.743
Body mass index, kg/m^2^	17.74 (16.02, 19.81)	17.78 (16.02, 19.85)	17.86 (16.04, 19.83)	17.58 (16.00, 19.52)	0.661
Calf, cm	24.0 (22.0, 26.0)	24.0 (22.5, 26.0)	24.5 (23.00, 26.7)	24.0 (22.0, 26.0)	0.001
Serum phosphate, mmol/L	1.07 (0.99, 1.18)	1.09 (1.01, 1.22)	1.08 (1.00, 1.18)	1.06 (0.98, 1.15)	0.010
Serum phosphate Categories					0.720
Low group [0.53, 0.89], %	74 (10.6)	18 (9.5)	23 (10.0)	33 (11.7)	
Normal group [0.89, 1.60], %	624 (89.0)	170 (89.9)	208 (90.0)	246 (87.6)	
High group [1.60, 2.33], %	3 (0.4)	1 (0.6)	0 (0.0)	2 (0.7)	
Creatine, µmol/L	74.00 (62.00, 90.00)	104.00 (96.00, 123.00)	77.00 (73.50, 82.00)	60.00 (53.00, 65.00)	˂0.001
eGFR	55.87 (44.13, 68.98)	37.28 (29.56, 41.46)	53.54 (49.33, 56.94)	70.67 (67.07, 74.15)	˂0.001
Testosterone, nmol/L	0.36 (0.16, 0.63)	0.39 (0.14, 0.69)	0.36 (0.17, 0.66)	0.33 (0.15, 0.61)	0.705
Testosterone Categories					0.630
Low group [0.08, 0.101], %	140 (20.0)	41 (21.7)	46 (19.9)	53 (18.9)	
Normal group [0.101, 1.42], %	536 (76.4)	140 (74.1)	180 (77.9)	216 (76.8)	
High group [1.42, 2.82], %	25(3.6)	8(4.2)	5(2.2)	12 (4.3)	
Estradiol, pmol/L	30.95 (18.40, 56.70)	37.90 (18.40, 64.50)	30.10 (18.40, 53.40)	30.90 (18.40, 51.60)	0.016
Estradiol Categories					
Normal group [18.4, 505], %	701 (100.0)	189 (100.0)	231 (100.0)	281 (100.0)	
Progesterone, nmol/L	0.61 (0.31, 0.97)	0.74 (0.40, 1.09)	0.61 (0.34, 0.96)	0.54 (0.27, 0.89)	0.001
Progesterone Categories					0.025
Low group [0.095-0.3], %	167 (23.8)	36 (19.0)	50 (21.6)	81 (28.8)	
Normal group [0.3–2.5], %	534 (76.2)	153 (81.0)	181 (78.4)	200 (71.2)	
Prolactin, ug/L	13.75 (10.57, 18.37)	14.21 (11.67, 19.49)	13.47 (10.61, 17.16)	13.75 (9.98, 18.46)	0.039
Prolactin Categories					0.570
Normal group [4.79, 23.3]	617 (88.0)	163 (86.2)	207 (89.6)	247 (87.9)	
High group [23.3, 45.9]	84 (12.0)	26 (13.8)	24 (10.4)	34 (12.1)	

All female centenarians had estradiol levels within the normal range. Progesterone levels were below the lower reference limit in 23.8% of participants, with the remainder falling within the normal range. Prolactin levels exceeded the upper reference limit in 12.0% of participants, with the remainder within the normal range. Testosterone levels were below or above the reference range in 20.0% and 3.6% of participants, respectively; the remainder had testosterone levels within the reference range. No significant differences in testosterone levels were observed among the CKD stage 1–2, stage 3a, and stage 3b–5 groups (*P* = 0.705). However, significant differences were observed for ethnicity (*P* = 0.049), calf circumference (*P* = 0.001), creatinine (*P* < 0.001), estradiol (*P* = 0.016), progesterone (*P* = 0.001), and prolactin (*P* = 0.039). Key indicators of mineral metabolism, namely serum phosphate levels, also differed significantly among the eGFR groups (*P* = 0.010), demonstrating an increasing trend as eGFR declined (eGFR ≥ 60 group: 1.06 mmol/L; eGFR < 45 group: 1.09 mmol/L).

**Fig. 2:**
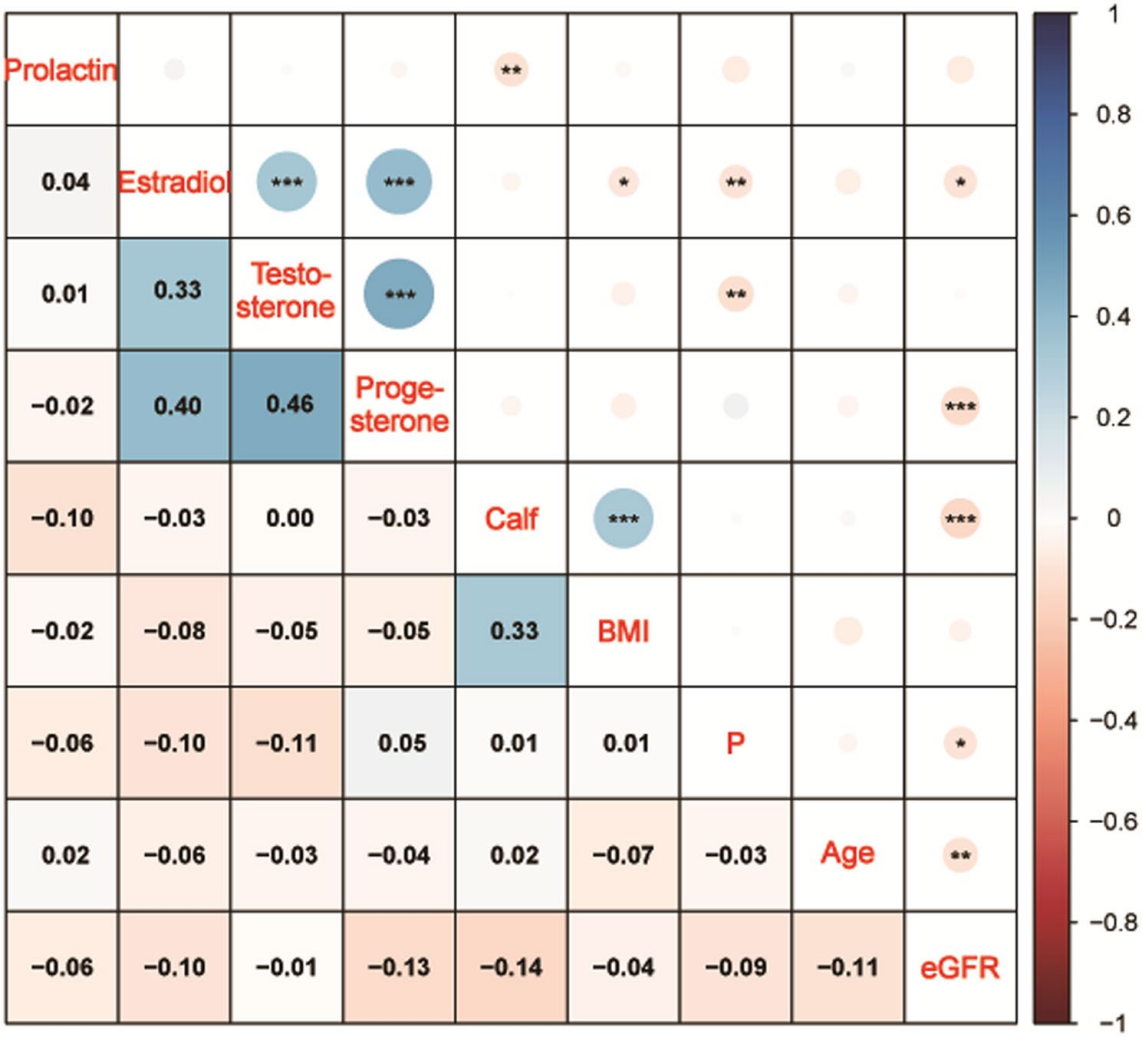
Correlation heatmap of baseline characteristics. The heatmap illustrates correlations among age, BMI, eGFR, calf circumference, serum phosphate (P), testosterone, estradiol, progesterone, and prolactin. The lower-left triangle displays correlation coefficients (r), ranging from − 1 to 1, with red indicating negative correlations and blue indicating positive correlations, and color intensity reflecting correlation magnitude. The upper-right triangle denotes the statistical significance of correlations: *P < 0.05, **P < 0.01, and ***P < 0.001. Correlation strength is defined as follows: |r| < 0.4 (weak), 0.4–0.7 (moderate), and |r| > 0.7 (strong)

Spearman correlation analysis of baseline data (Fig. 2) revealed significant positive correlations among serum sex hormone levels. Estradiol was weakly positively correlated with testosterone (*r* = 0.33, *P* < 0.001), progesterone was moderately positively correlated with testosterone (*r* = 0.46, *P* < 0.001) and estradiol (*r* = 0.40, *P* < 0.001). Additionally, among anthropometric indicators, calf circumference was weakly positively correlated with BMI (*r* = 0.33, *P* < 0.001).

### Association between eGFR and all-cause mortality

**Fig. 3:**
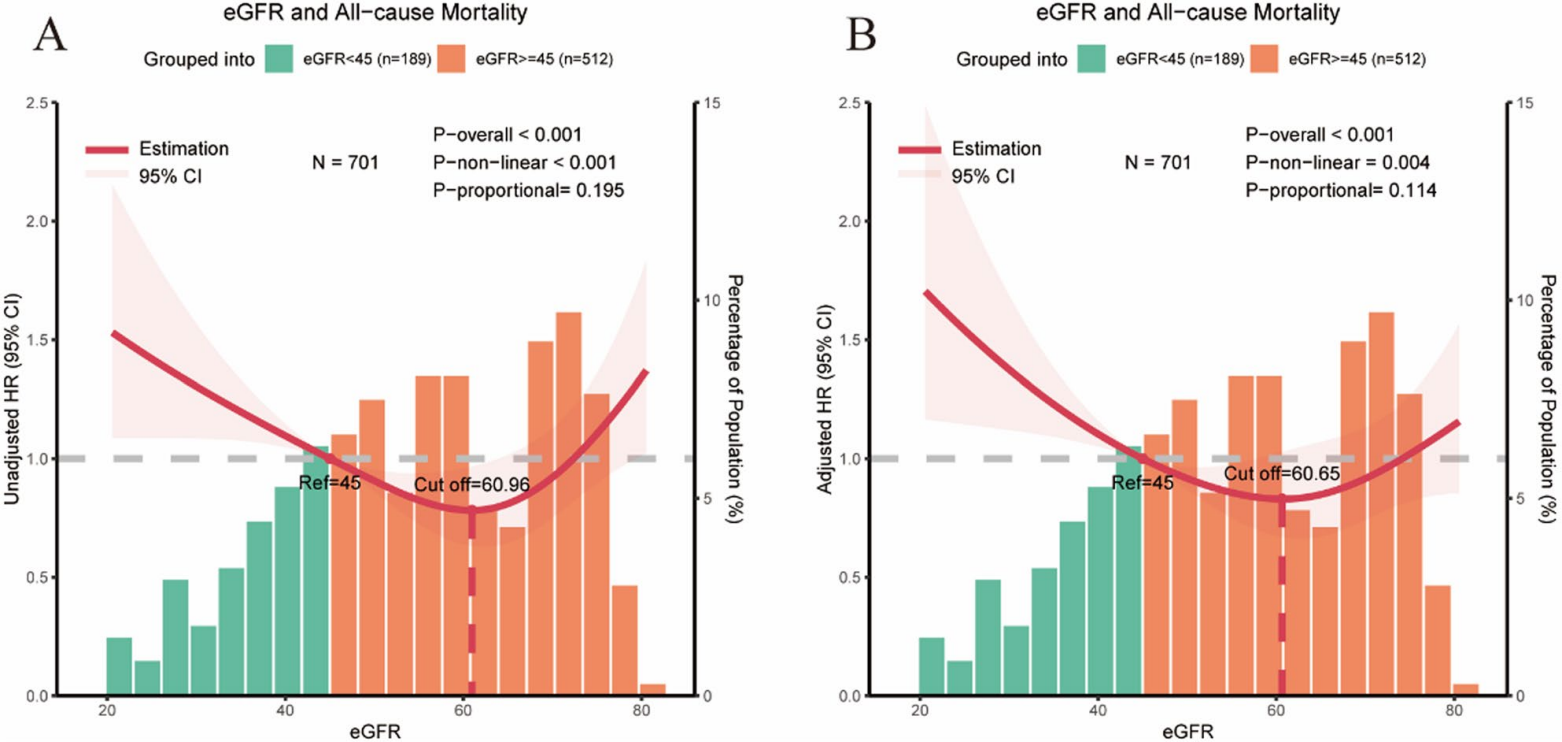
RCS analysis of the association between eGFR and all-cause mortality. The solid red line represents the HR, and the shaded area indicates the 95% CI. The horizontal axis shows eGFR values; the left vertical axis shows HRs for all-cause mortality; and the right vertical axis shows the percentage distribution of centenarian participants across eGFR values. Panel A presents the univariable analysis, and panel B presents the multivariable analysis adjusted for age, ethnicity, marital status, body mass index, education level, smoking status, alcohol consumption, diabetes mellitus, hypertension, coronary heart disease, calf circumference, serum phosphate, and sex hormones (testosterone, estradiol, progesterone, and prolactin). The reference eGFR value was set at 45 mL/min/1.73 m². A U-shaped association between eGFR and all-cause mortality was observed in both the unadjusted (P-overall < 0.001; P-nonlinear < 0.001) and multivariable-adjusted analyses (P-overall < 0.001; P-nonlinear = 0.004). The inflection point of eGFR identified in the adjusted RCS model was 60.65 mL/min/1.73 m²

RCS analysis (Fig. 3) revealed a significant nonlinear association between eGFR and the risk of all-cause mortality in the unadjusted model (*P* < 0.001; P-nonlinear < 0.001) (Fig. 3A). The relationship exhibited a distinct U-shaped pattern with an inflection point at 60.96 mL/min/1.73 m². Specifically, eGFR levels below this threshold were associated with an increased risk of mortality as eGFR declined, while levels above 60.96 mL/min/1.73 m² were associated with increased risk as eGFR increased. This nonlinear relationship remained significant after adjustment for potential confounders (*P* <0.001; P-nonlinear = 0.004) (Fig. 3B), with the inflection point shifting slightly to 60.65 mL/min/1.73 m². The proportional hazards assumption was subsequently evaluated. Test results for proportionality (unadjusted model: *P* = 0.195; adjusted model: *P* = 0.114) indicated no statistically significant violation of the assumption, confirming that application of the Cox proportional hazards model was appropriate.

To verify the robustness of conclusions derived from the CKD-EPI 2009 equation, analyses were repeated using five alternative equations: BIS1, FAS, RLM, EKFC, and CKD-EPI 2021. Consistent with the primary analysis, RCS analyses (Figure S1) for all five equations revealed significant overall associations (all the *P* < 0.001) and distinct nonlinear patterns (all the P-nonlinear < 0.05) in both unadjusted and multivariable-adjusted models. Sensitivity analyses were additionally performed by excluding participants with extreme eGFR values (< 15 or > 90 mL/min/1.73 m²) or those who died within the first 30 days of follow-up. The results (Figure S2) remained consistent, with statistically significant overall associations (all the *P* < 0.05) and nonlinearity (all the P-nonlinear < 0.05), confirming the robustness of the findings.

To further validate the stability of the eGFR-all-cause mortality association, we performed sensitivity analyses using restricted cubic spline (RCS) interaction models to assess the modifying effects of serum phosphate, calf circumference, and BMI (Figure S3). In both unadjusted and multivariable-adjusted models, none of the interaction P-values reached statistical significance (serum phosphate: 0.962 and 0.746; calf circumference: 0.260 and 0.118; BMI: 0.510 and 0.779; all *P* > 0.05). These results confirm that the U-shaped eGFR-mortality association and testosterone’s modifying effect are robust and not confounded by these factors.

As shown in Table 2, univariable Cox proportional hazards regression analysis indicated that each 10-mL/min/1.73 m² increase in eGFR was associated with a 5.1% reduction in all-cause mortality risk (HR = 0.949, 95% CI: 0.901–1.001; *P* = 0.054). After multivariable adjustment, each 10-mL/min/1.73 m² increase in eGFR was associated with a 6.8% reduction in all-cause mortality risk (adjusted HR = 0.932, 95% CI: 0.882–0.985; *P* = 0.012). The group with 45 ≤ eGFR < 60 mL/min/1.73 m², which exhibited the lowest observed all-cause mortality, was used as the reference group. Compared with this reference group, female centenarians with eGFRs of < 45 mL/min/1.73 m² had a significantly increased risk of all-cause mortality (unadjusted HR = 1.499, 95% CI: 1.227–1.831, *P* < 0.001; adjusted HR = 1.449, 95% CI: 1.176–1.785, *P* < 0.001). By contrast, no statistically significant difference in all-cause mortality risk was observed for female centenarians with eGFRs of ≥ 60 mL/min/1.73 m² compared with the reference group in either univariable or multivariable analyses. Overall, multivariable analysis indicated that female centenarians with eGFRs of < 45 mL/min/1.73 m² had a 44.9% higher risk of all-cause mortality than the reference group.

**Table 2:** Cox regression analyses of associations of eGFR with all-cause mortality in female centenarians

Terms	Count	Univariate analysis		Multivariate -adjusted analysis	
eGFR	701	HR	*P*	HR	*P*
		Continuous, per 10 ml/min/1.73m^2^ 0.949(0.901,1.001)	0.054	0.932(0.882,0.985)	0.012
Grouped by Clinical Value					
eGFR < 45	189	1.499(1.227,1.831)	< 0.001	1.449(1.176,1.785)	< 0.001
45 ≤ eGFR < 60	231	1 (Reference)		1 (Reference)	
eGFR ≥ 60	281	1.140(0.948,1.370)	0.165	1.085(0.898,1.312)	0.398
*P* for trend			0.017		0.014

The data were adjusted for demographic factors (age, ethnicity, marital status and education), body mass index, lifestyle factors (cigarette smoking and alcohol consumption), comorbidities (hypertension, diabetes mellitus, and coronary heart disease), calf circumference, serum phosphate, and sex hormones (testosterone, estradiol, progesterone, and prolactin)

**Fig. 4:**
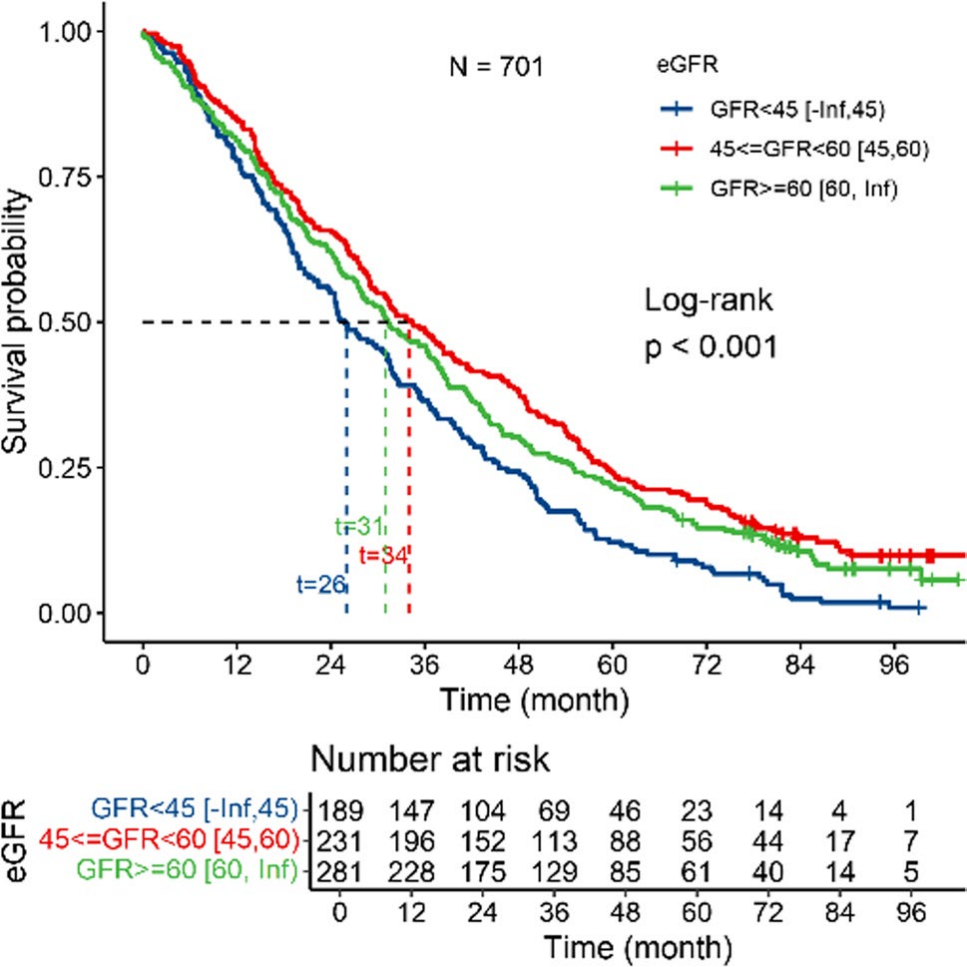
Kaplan–Meier survival curves and log-rank tests for all-cause mortality according to eGFR categories. Kaplan–Meier survival analysis demonstrated a significant association between lower eGFR and shorter survival time. Participants with eGFR values of 45–60 mL/min/1.73 m² exhibited the longest median survival time compared with those with eGFR < 45 mL/min/1.73 m² and eGFR ≥ 60 mL/min/1.73 m² (34 months vs. 26 months vs. 31 months, respectively; log-rank *P* < 0.001)

Kaplan–Meier survival analysis demonstrated that lower eGFR levels were significantly associated with higher all-cause mortality (log-rank test, *P* < 0.001) (Fig. 4). Among the female centenarians, those with eGFRs of < 45 mL/min/1.73 m² (CKD stage 3b–5) had a significantly shorter median survival time (26 months) than those with 45 ≤ eGFR < 60 mL/min/1.73 m² (CKD stage 3a; 34 months; log-rank *P* < 0.001).

### Specificity of the effects of sex hormones

**Fig. 5:**
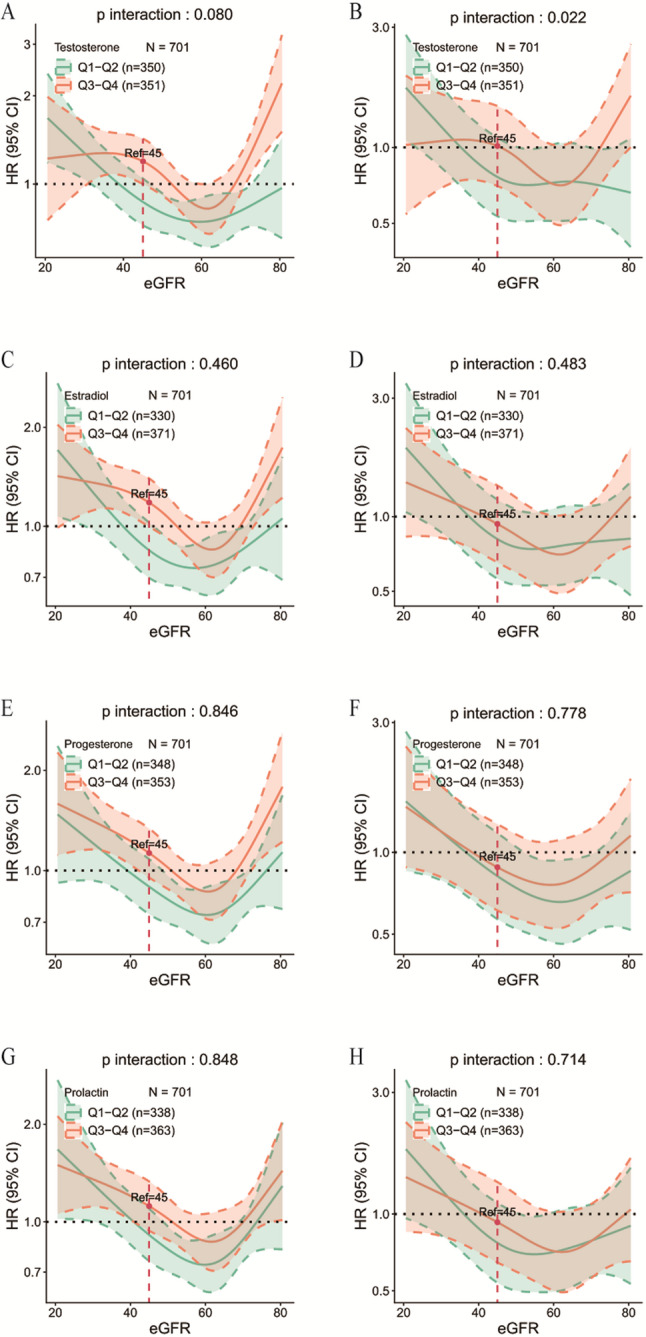
Effects of sex hormones on the association between eGFR and all-cause mortality in female centenarians. Unadjusted and multivariable-adjusted RCS interaction analyses illustrate the modifying effects of different sex hormone levels on the relationship between eGFR and mortality risk. Panels **A** and **B** show the interaction between testosterone levels and eGFR in the unadjusted and multivariable-adjusted models, respectively. Panels **C** and **D** depict estradiol, panels **E** and **F** progesterone, and panels **G** and **H** prolactin. Significant nonlinear interaction effects were observed only for testosterone (nonlinear unadjusted *P* = 0.080; nonlinear multivariable-adjusted *P* = 0.022), whereas no significant interaction effects were identified for estradiol, progesterone, or prolactin

To clarify the specific influence of sex hormones on the eGFR–mortality association, RCS models were further employed to assess nonlinear interactions (Fig. 5). Within the multivariable-adjusted model, a significant nonlinear interaction was observed between testosterone levels and eGFR (P-nonlinear interaction = 0.022), confirming an independent role of testosterone in modifying the predictive association between eGFR and mortality risk.

The RCS curves revealed distinct patterns:

- Among female centenarians with low testosterone levels (< 0.36 nmol/L) and eGFRs of < 45 mL/min/1.73 m², a steep and significant inverse relationship persisted between eGFR and all-cause mortality, with mortality risk increasing sharply as eGFR declined.
- By contrast, among those with high testosterone levels (≥ 0.36 nmol/L), all-cause mortality increased only slightly as eGFR declined below 45 mL/min/1.73 m², resulting in an attenuated association.

These findings suggest that higher testosterone levels may mitigate the increased mortality risk associated with low eGFR (< 45 mL/min/1.73 m²), indicating a potential effect modification (univariate nonlinear fit: *P* = 0.080; multivariable nonlinear fit: *P* = 0.022) (Fig. 5A, B). No significant nonlinear interaction effects were observed for estradiol, progesterone, or prolactin (all P-nonlinear interactions > 0.05) (Fig. 5C–H).

## Discussion

This study is the first to systematically elucidate the association between eGFR and all-cause mortality in an extremely long-lived cohort of Chinese female centenarians, while also revealing the key regulatory role of serum testosterone levels in this relationship. Our core findings demonstrate a significant U-shaped nonlinear association between eGFR and all-cause mortality, identifying 45 and 60 mL/min/1.73 m² as critical inflection points for predicting survival prognosis. More importantly, we found that higher physiological testosterone levels significantly attenuate the mortality risk driven by declining eGFR. This discovery provides a new theoretical basis for renal health assessment and personalized intervention in extremely aged populations.

Regarding the association pattern between eGFR and mortality, this study established a U-shaped relationship among female centenarians, with the 45–60 mL/min/1.73 m² range corresponding to the lowest mortality risk. This finding is highly consistent with epidemiological evidence across different populations. For instance, a nationwide study in South Korea covering nearly 10 million individuals confirmed that both low eGFR (< 60 mL/min/1.73 m²) and extremely high eGFR (≥ 120 mL/min/1.73 m²) are independently associated with increased all-cause mortality due to cardiovascular disease, cancer, and infection [9]. Similarly, a meta-analysis including 114 global cohorts reported a comparable U-shaped association in the general population [4]. By extending this pattern to the centenarian subgroup, this study validates the universal applicability of the nonlinear relationship between renal function and survival outcomes across the entire life cycle. Crucially, the inflection point of 45 mL/min/1.73 m² identified in this specific U-shaped relationship lends strong support to the recommendation from the 2019 international expert consensus regarding age-adapted thresholds, specifically lowering the CKD diagnostic criterion to < 45 mL/min/1.73 m² for individuals aged ≥ 65 years [10]. This threshold may represent the functional reserve limit of the kidneys required to maintain survival under conditions of extreme aging.

On the right side of the U-shaped curve, the increased mortality risk observed in the higher eGFR group (≥ 60 mL/min/1.73 m²) must be interpreted with caution from both methodological and pathophysiological perspectives. Multiple lines of evidence suggest that falsely elevated eGFR values in the elderly often do not represent true renal hyperfiltration but rather reflect sarcopenia and malnutrition. A Finnish study noted that eGFR standardized to a body surface area of 1.73 m² overestimates renal function in smaller individuals; when corrected for actual body surface area, the association between hyperfiltration and mortality disappeared [11]. Given that the median BMI of this study cohort was only 17.74 kg/m² (below the WHO standard for normal weight), the high prevalence of sarcopenia and malnutrition likely led to reduced creatinine generation, resulting in an artifactual elevation of creatinine-based eGFR [12]. This mechanism explains why high eGFR is associated with poor prognosis: it fundamentally reflects severe muscle wasting and frailty rather than pathological renal changes. This interpretation is consistent with findings from a Korean study observing an association between high eGFR and cancer mortality [9], as well as a study in the Chinese population showing no survival benefit for eGFR > 90 mL/min/1.73 m² [13], suggesting that the increased risk in this range is primarily mediated by non-renal factors.

The most novel finding of this study lies in the significant moderating effect of serum testosterone on the eGFR–mortality association. Although previous meta-analyses in patients with CKD have demonstrated that low testosterone is closely associated with increased all-cause mortality (HR: 1.98) and cardiovascular events (HR: 2.40) [14], such evidence has predominantly focused on males. This study is the first to confirm that in female centenarians, higher physiological testosterone levels exert a crucial survival-protective effect, highlighting cross-gender beneficial effects of testosterone on kidney-related prognosis.

The potential biological mechanisms underlying this protective effect involve multiple pathways. First, testosterone contributes to the maintenance of muscle mass and metabolic homeostasis. Although often characterized as a male hormone, testosterone—derived from the adrenal glands and ovaries—is essential for maintaining skeletal muscle synthesis in females. Alexander et al. proposed that testosterone is positively correlated with muscle mass in both premenopausal and postmenopausal women [15]. Given that low muscle mass is an independent risk factor for mortality in the elderly [16–18], higher testosterone levels may reduce mortality risk by mitigating the progression of sarcopenia and improving functional status [19]. Second, testosterone may improve vascular endothelial function and lipid metabolic profiles. Evidence suggests a positive correlation between endogenous testosterone levels and endothelial function in postmenopausal women. Rech et al. found that low testosterone levels were significantly associated with endothelial dysfunction in early postmenopausal women [20]. Complementing these findings, a recent study involving women over 70 years of age provided evidence for a potential cardiovascular protective role of testosterone in older women, demonstrating that higher endogenous testosterone levels are independently associated with a more favorable lipid profile, characterized by higher high-density lipoprotein cholesterol and lower triglycerides [21]. Testosterone may directly improve microcirculation through regulation of vascular tone and the exertion of anti-inflammatory and antioxidant effects, thereby counteracting the cardiovascular burden associated with declining renal function. Third, hormonal transformation and balance may play an important role. In female centenarians, the metabolic kinetics of testosterone are altered. Following menopause, peripheral conversion of androgen precursors (such as dehydroepiandrosterone) becomes the primary source of circulating testosterone [22]. Concurrently, testosterone serves as a substrate for aromatase-mediated conversion to estradiol in tissues such as adipose tissue; this conversion is important for maintaining hormonal balance under conditions of extremely low postmenopausal estrogen. Although high-level evidence has confirmed the protective effects of estradiol on skeletal and cardiovascular systems in early postmenopausal women, the overall metabolic health of postmenopausal women relies on a more complex endocrine balance, driven by peripheral tissue conversion and involving coordinated regulation by testosterone and estradiol [23].

Our findings also help clarify previous controversies regarding the relationship between testosterone and mortality in women. Unlike the positive correlation reported by Wang et al. [24], whose study participants were younger (approximately 60 years old) and had higher baseline testosterone levels, studies of postmenopausal women with diabetes have shown that low testosterone increases mortality risk [25], which aligns with our conclusions. This discrepancy suggests a window effect for testosterone action. Testosterone levels in female centenarians (median: 0.36 nmol/L) are substantially lower than those in younger postmenopausal women and fall within a lower physiological range. Within this range, moderately elevated testosterone levels may compensate for age-related anabolic deficits without producing the adverse effects associated with supraphysiological exposure.

Finally, this study did not identify significant moderating effects of estradiol, progesterone, or prolactin on the association between eGFR and mortality. This may be attributable to the extremely low estradiol levels observed in centenarians (median: 30.95 pmol/L), which likely fall below the threshold required to exert systemic regulatory effects. In addition, the physiological roles of progesterone and prolactin are largely limited to reproductive regulation and may have weaker associations with the key metabolic and renal processes essential for survival in advanced age [26].

This study has several limitations. First, eGFR was calculated solely using baseline serum creatinine levels, precluding assessment of dynamic changes in renal function during follow-up. Second, associations between eGFR and cause-specific mortality, such as cardiovascular or cerebrovascular mortality, were not examined. Third, although the optimal eGFR estimation equation for older adults remains debated, the latest Chinese Expert Consensus on the Diagnosis and Management of Chronic Kidney Disease in the Elderly recommends the CKD-EPI creatinine equation and notes that the CKD-EPI creatinine–cystatin C equation may provide greater accuracy, cystatin C measurements were not available in our cohort. Fourth, the sample size was relatively small and consisted exclusively of female centenarians from Hainan, China, limiting the generalizability of the findings to other populations or ethnic groups. Finally, the molecular mechanisms underlying the observed associations remain to be elucidated in future studies.

## Conclusion

This study is the first to investigate the influence of sex hormones, particularly testosterone, on the relationship between eGFR and all-cause mortality among female centenarians in Hainan, China. A significant nonlinear (U-shaped) association between eGFR and all-cause mortality was observed. Importantly, testosterone levels modified this association: low testosterone amplified the adverse effect of reduced eGFR on mortality, whereas higher testosterone levels attenuated the mortality risk associated with declining eGFR, indicating a protective effect. These findings suggest that testosterone may represent a potential therapeutic target for mitigating aging-related disease risk, particularly in individuals with impaired renal function. Further studies are warranted to elucidate the underlying mechanisms by which sex hormones modify the link between eGFR and mortality.

## Supplementary Information

Supplementary Material 1.

## Data Availability

The data that support the findings of this study are available from the corresponding author upon reasonable request.
