# Supplementary material for: Testosterone modifies U-Shaped association of eGFR with all-cause mortality in Chinese female centenarians: a prospective cohort study
Source: Reprod Biol Endocrinol. 2026 Jan 28;24:31. doi: 10.1186/s12958-026-01529-w (PMC12924263; doi:10.1186/s12958-026-01529-w)
Supplement: Supplementary file 1 — Supplementary Material 1. [file 12958_2026_1529_MOESM1_ESM.docx]

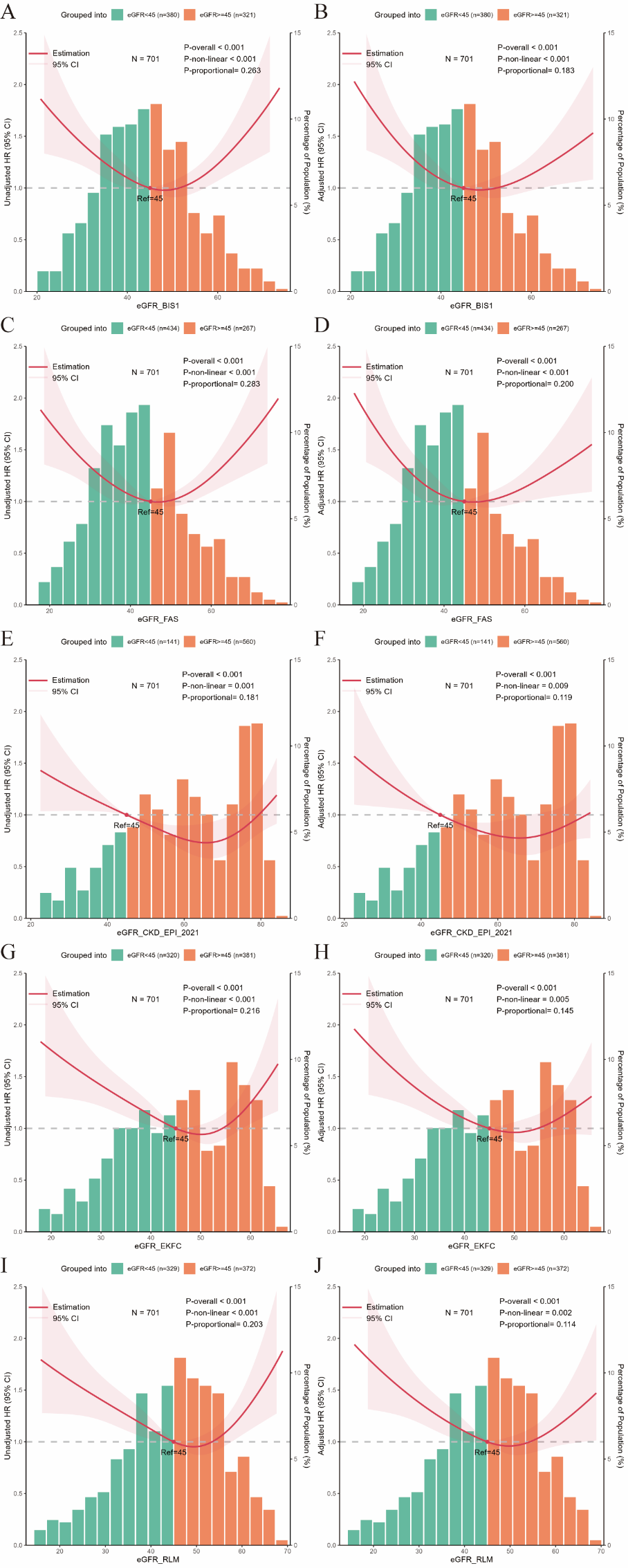


(See figure on previous page.)

**Figure S1. RCS analyses of the association between eGFR and all-cause mortality using different estimation equations**

The associations between eGFR and all-cause mortality were significantly nonlinear in both unadjusted (A, C, E, G, I) and multivariable-adjusted (B, D, F, H, J) RCS models.

A, B: Calculated using the Berlin Initiative Study (BIS1) creatinine-based equation

C, D: Calculated using the Full Age Spectrum (FAS) creatinine-based equation

E, F: Calculated using the Chronic Kidney Disease Epidemiology Collaboration 2021 (CKD-EPI 2021) creatinine-based equation

G, H: Calculated using the European Kidney Function Consortium (EKFC) creatinine-based equation

I, J: Calculated using the revised Lund–Malmö (RLM) creatinine-based equation


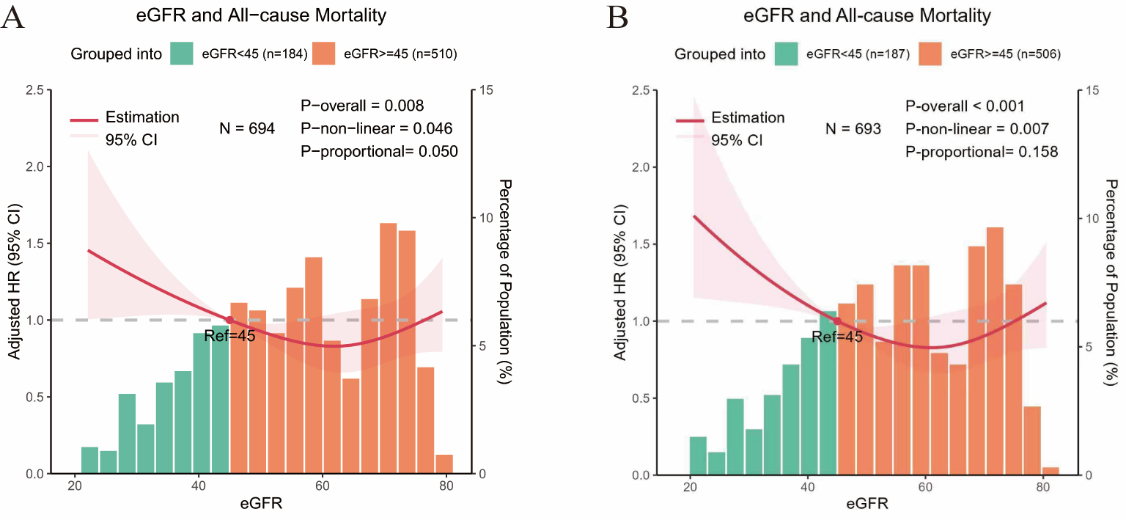


**Figure S2. Multivariable-adjusted RCS analyses of the association between eGFR and all-cause mortality**

A: Excluded centenarians with eGFR of <15 or >90 mL/min/1.73 m² (n = 694)

B: Excluded centenarians who died within 30 days after the start of follow-up (n = 693)


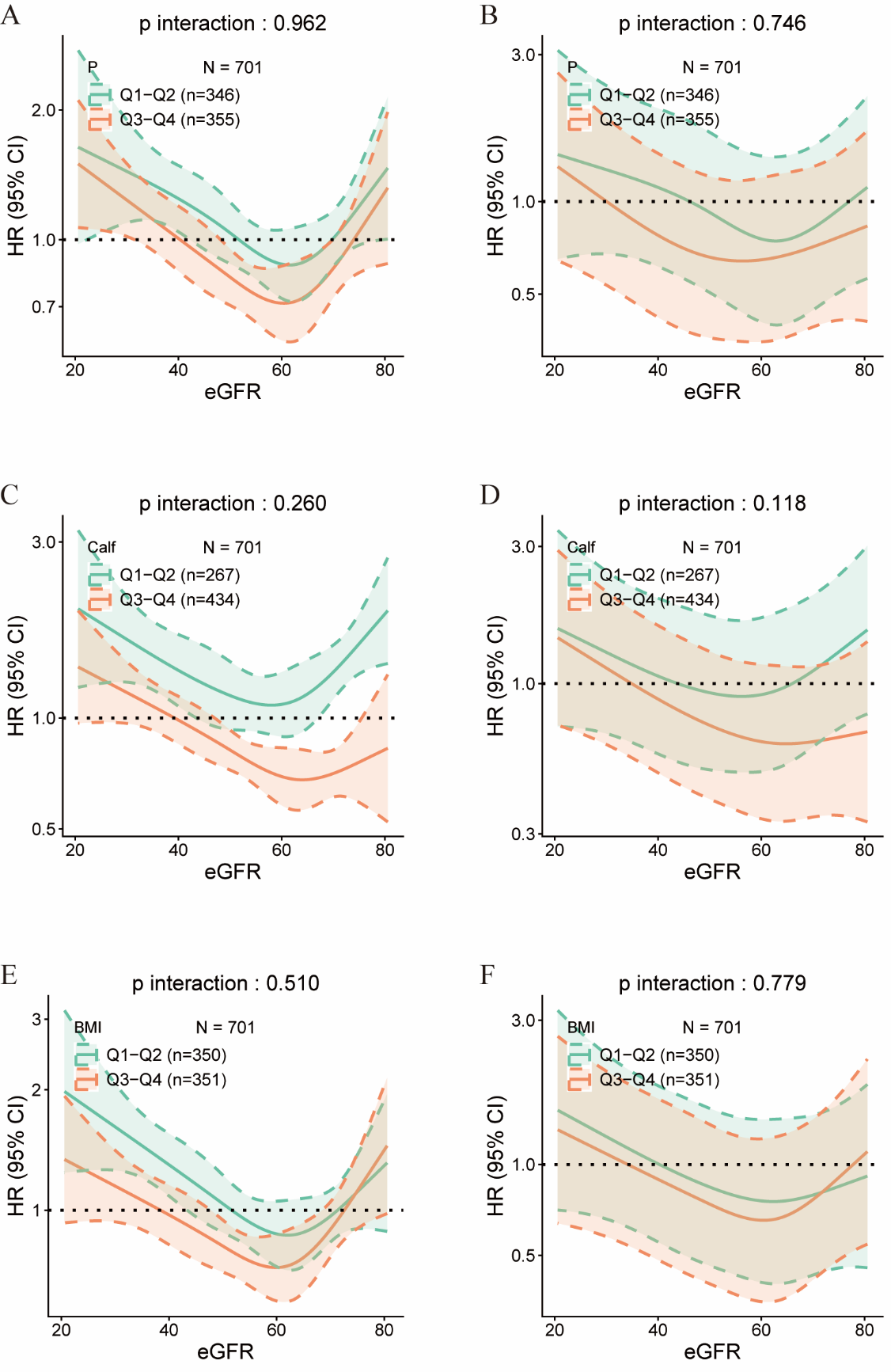


**Figure S3. Effects of other factors on the association between eGFR and all-cause mortality in female centenarians.** Unadjusted and multivariable-adjusted RCS interaction analyses illustrate the modifying effects of serum phosphate, calf circumference and BMI on the relationship between eGFR and mortality risk. Panels A and B show the interaction between serum phosphate and eGFR in the unadjusted and multivariable-adjusted models, respectively. Panels C and D depict calf circumference, panels E and F BMI. No significant interaction effects were found for serum phosphate, calf circumference or BMI.
